# Graphene Oxide Membranes for Sustainable Recycling: Poly(styrene) Fractionation by Organic Solvent Nanofiltration

**DOI:** 10.1021/acsengineeringau.5c00102

**Published:** 2025-12-31

**Authors:** Natechanok Yutthasaksunthorn, Yuchen Chang, Van Son Nguyen, Kaung Su Khin Zaw, Scott A. Sinquefield, Carsten Sievers, Sankar Nair

**Affiliations:** † School of Chemical & Biomolecular Engineering, 1372 Georgia Institute of Technology, Atlanta, Georgia 30332, United States; ‡ Renewable Bioproducts Institute, 1372 Georgia Institute of Technology, Atlanta, Georgia 30332, United States

**Keywords:** Graphene Oxide Membranes, Plastic Recycling, Poly(styrene), Fractionation, Nanofiltration, Depolymerization

## Abstract

Efficient separation and purification of polymeric mixtures is an important challenge in plastic recycling. Here we demonstrate a robust graphene oxide (GO) membrane platform capable of separating low- and high-molecular-weight poly­(styrene) (PS) in nonpolar solvents. By tuning GO membrane properties through pillaring with a polyconjugated aromatic compound (PAC) and controlled reduction, we obtain the efficient nanofiltration of poly­(styrene) in a hydrocarbon solvent, enabling the removal of monomers and low-molecular-weight oligomers. Over 600 h of continuous operation, the pillared membrane maintains a stable high flux of 8 ± 1 L m^–2^ h^–1^ and total rejection of high-MW polymer. Postfractionation, the enriched high-MW retentate has a 2-fold higher yield of styrene monomers in mechanocatalytic ball-milling depolymerization compared to unfractionated PS. Removing oligomeric diluents improves energy transfer, suppresses chain transfer, and promotes chain scission followed by chain-end depropagation. Thus, fractionation by organic solvent nanofiltration with GO membranes can enable scalable and efficient routes to mechanochemical polymer recycling.

## Graphical abstract

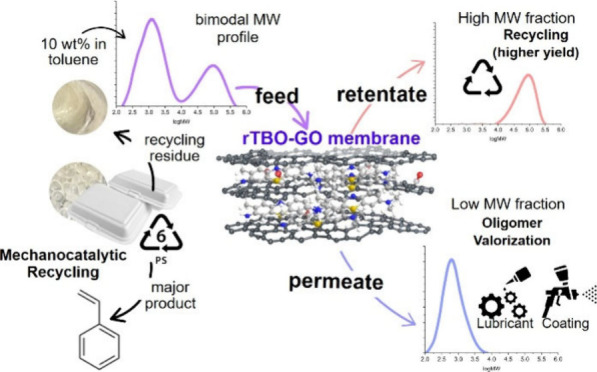

The accumulation of plastic waste, especially from polyolefins, presents a growing environmental and economic challenge as global efforts turn toward circular material strategies. Among these, poly­(styrene) (PS) remains one of the difficult polymers to recycle effectively, in part due to the chemically diverse and heterogeneous mixtures produced during thermal, catalytic, or mechanochemical depolymerization. Plastic waste streams often contain both low-MW and high-MW components, which complicates downstream processing and significantly reduces monomer recovery efficiency.^1–4^ A key barrier to closed-loop recycling is the coexistence of low-MW oligomers in feed streams and partially depolymerized residues. These small-chain species can interfere with further depolymerization reactions by deactivating catalysts, absorbing energy, or altering local chemical equilibria.^5^ Additionally, the absence of scalable separation methods has limited the reuse of partially depolymerized residues.

Conventional purification approaches, such as distillation or solvent extraction, are energy-intensive and poorly suited for high-viscosity polymer mixtures.^6,7^ Membrane-based separation technologies, particularly organic solvent nanofiltration (OSN), have emerged as energy-efficient alternatives for separating molecules in organic streams. Figure 1 depicts a process in which an OSN membrane integrated downstream of mechanocatalytic depolymerization fractionates the recycling residue mixture of low-MW oligomers and high-MW polymer into a low-MW, oligomer-rich permeate (sent to solvent recovery and further valorization) and a high-MW retentate recycled to the reactor. Toluene is introduced at dilution, recovered by distillation, and recycled to the same node, thereby closing both polymer and solvent loops.

**Figure 1:**
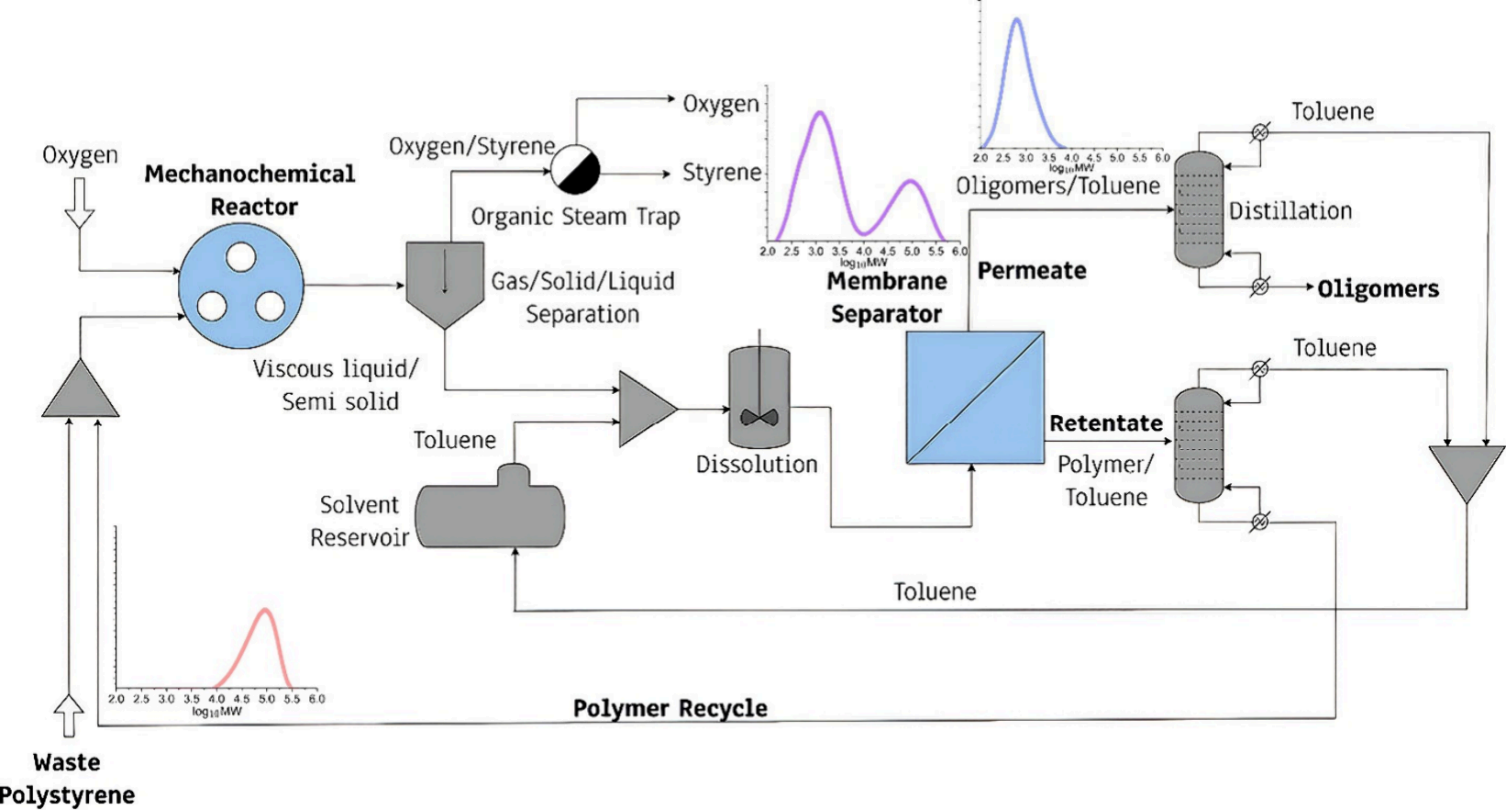
Illustration of a membrane-enabled process for mechanochemical depolymerization of poly­(styrene).

While numerous OSN membranes have been developed, most are specifically designed for the separation of mixtures in polar organic solvents such as methanol, ethanol, and acetone.^8–10^ In contrast, there are relatively few membranes that have been utilized for the separation of mixtures in hydrocarbon solvents such as hexane, and toluene.^11–13^ According to our recent discussion,^14^ state-of-the-art polymeric OSN membranes exhibit a wide range of solvent permeances of 0.1–20 L m^–2^ h^–1^ bar^–1^. Yet, most commercial OSN membranes still face issues of swelling, plasticization, and restricted solvent selection, especially in nonpolar solvents. This is a major limitation, as nonpolar solvents are widely used in plastics recycling due to their compatibility with hydrocarbon-based polymers like PS.^15,16^ The few prior nonpolar OSN studies in realistic streams largely target purification (contaminant removal or polishing)^17,18^ rather than fractionation of concentrated depolymerization streams. Improving membrane performance in nonpolar media requires a significant shift in membrane design. Recent OSN work has focused on intrinsically hydrophobic polymer membranes, including polyketone composites, tuned polymer films, and fluorinated polymer architectures designed to resist swelling and plasticization in aromatics and alkanes.^19–22^ Despite these advances, long-term stability in mixed, highly nonpolar feeds and scalable manufacture remain open challenges, particularly relative to the solvent-compatibility limits of current commercial OSN polymers.^23,24^

Graphene oxide (GO)-based membranes offer a promising platform to address these challenges. Their unique two-dimensional (2D) lamellar architecture provides highly tunable interlayer spaces for size-selective transport, while the abundance of functional groups enables precise chemical modification.^24–29^ Moreover, GO sheets can be assembled into continuous, defect-free films with subnanometer control over porosity, which is key for molecular discrimination in nanofiltration and scale-up processes.^30^ Importantly, while pristine graphene oxide (GO) membranes are inherently hydrophilic due to the oxygenated functional groups,^31,32^ the membranes can be chemically or thermally reduced in order to make them more organophilic. However, reduction also leads to progressive collapse of the interlayer spaces (i.e., graphitization), severely limiting permeability.

To overcome these constraints, we recently demonstrated GO membranes intercalated with polyconjugated aromatic compounds (PACs) to pillar the interlayer spaces, followed by controlled chemical reduction using hydriodic acid (HI).^14^ This dual modification strategy enhances membrane hydrophobicity while stabilizing the interlayer spaces. Specifically, reduced GO membranes pillared with toluidine blue O (TBO) and solvent green (SG) showed high permeabilities in a broad spectrum of nonpolar alkane and aromatic solvents. Furthermore, these membranes crossflow nanofiltration separation of low-MW and high-MW solutes in toluene solvent. The composition (degree of reduction, intercalant distribution) and microstructure (interlayer spacings, pore size distributions) were characterized in detail.^14^ In the above context, the goal of this Letter is to study the characteristics of our recently developed pillared and reduced GO membranes to fractionate realistic PS streams relevant to the process shown in Figure 1. Here, we use industrially available PS pellets with a broad MW distribution as a realistic representative of a partially depolymerized PS stream.^33^ We use toluene as a good solvent for poly­(styrene), and it can also be easily recovered by distillation.^34,35^ We demonstrate the fractionation of this feed stream in crossflow operation exceeding 600 h, and analyze the characteristics of the retentate and permeate streams in detail. Furthermore, we show the dramatically increased mechanocatalytic depolymerization efficiency of the membrane-fractionated PS feedstock relative to the unfractionated PS feedstock. The scalable, stable nanostructured nonpolymeric membrane platform for fractionation of depolymerized plastic residues allows enhanced efficiency of poly­(styrene) recycling.

Four distinct types of rGO membranes were prepared and evaluated for their ability to fractionate poly­(styrene) (PS) solutions. These membranes are pillared with π-conjugated aromatic molecules, namely (7-amino-8-methyl­pheno­thiazin-3-ylidene)-dimethyl­ammo­nium chloride (also known as Toluidine Blue O, “TBO”) or trisodium 8-hydroxy­pyrene-1,3,6-trisulf­onate (also known as Solvent Green 7, “SG”). They are subsequently reduced with 5.7 wt % HI (see Experimental Methods in the Supporting Information (SI)), yielding rTBO-GO and rSG-GO membranes, respectively. Two nonpillared rGO membranes were prepared, reduced using aqueous concentrations of 5.7 and 22.8 wt % to obtain different degrees of reduction (as quantified by the C/O ratio obtained by XPS), and are labeled as rGO1 and rGO2. Key structure–property comparisons (C/O, intercalant/loading, reduction, *d*-spacing; rejection and permeance) are summarized in Tables S1–S3 (SI). Photographs of the membrane coupons (47 mm in diameter) are presented in Figure S1, highlighting visual differences associated with their distinct chemical compositions and fabrication routes. To ensure sufficient feed volume and reproducibility in membrane fractionation experiments, commercially available poly­(styrene) (PS) pellets exhibiting a bimodal molecular weight (MW) distribution were dissolved (10 wt %) in toluene and used as a realistic representative of depolymerized PS residues obtained from mechanocatalytic processing. Figures 2a–2c show a sample of mechanochemically depolymerized PS prepared by methods shown recently,^33^ its dissolution in toluene, and its bimodal MW distribution. The depolymerized PS exhibits a broad distribution dominated by low-MW oligomers and monomers (log_10_MW 2.2–4, ∼150–10,000 g/mol), and shows a high-MW distribution extending up to ∼180,000 g/mol. Figures 2d–2f show the PS pellets, their dissolution in toluene, and the MW distribution. This 10 wt % feed solution is a good representative of depolymerized PS, can be prepared in sufficient quantities, and is similar to practical feeds in solvent-based plastic recycling processes.^33,36^

**Figure 2:**
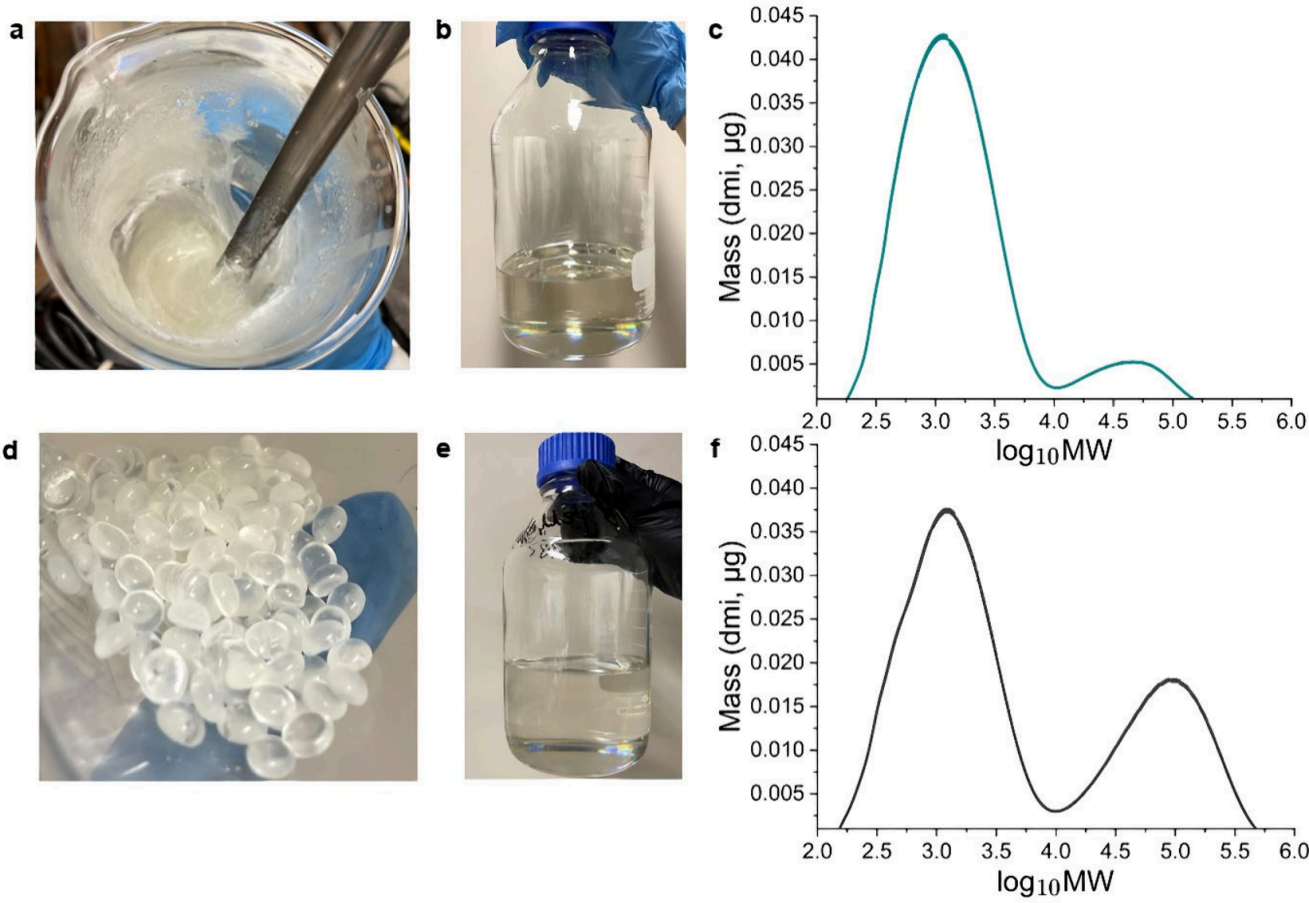
(a–c) Mechanocatalytically depolymerized PS residue prepared according to ref 33: (a) viscous liquid-semisolid form, (b) 10 wt % solution in toluene, and (c) molecular weight distribution. (d–f) Commercial PS pellets as representative feed: (d) pellets, (e) 10 wt % solution in toluene, and (f) molecular weight distribution.

Figures 3a–3h summarize the results of crossflow nanofiltration measurements with the four membranes, conducted with the apparatus of Figure S2. The nonpillared rGO membranes (rGO1 and rGO2) completely rejected the high-MW fraction but showed other distinct differences. The greater degree of reduction/deoxygenation in rGO2 is associated with greater collapse of the interlayer spaces, leading to a higher rejection of the low-MW fraction (Figures 3a, 3b, 3g). and lower fluxes and permeance (Figures 3e, 3f, 3h) than the rGO1 membrane. The low-MW fraction window is ∼100–10,000 g/mol (log_10_MW 2–4), and the high-MW fraction region is ∼10,000–1,000,000 g/mol (log_10_MW 4–6) as derived from GPC-calibrated PS standards. The rejection and permeance data at 10–30 bar are provided in Tables S2 and S3. The two pillared membranes (rTBO-GO and rSG-GO) also completely rejected the high-MW fraction, but the pillared rTBO-GO membrane has more favorable characteristics over the rSG-GO membranes in terms of higher flux and permeance, and lower rejection of the low-MW fraction. Both pillared membranes were fabricated with a reduction treatment identical to rGO1. As we have shown in detail recently,^14^ the rTBO-GO membrane (containing 27 wt % TBO) has a high concentration of TBO h-dimers and h-trimers (i.e., stacked TBO molecular arrangements) pillaring the interlayer spaces. In contrast, the rSG-GO (with 32 wt % SG) membrane does not exhibit dimer formation, and the intercalant molecules are distributed in lateral arrangements only. As a result, the rTBO-GO membrane has a much more expanded interlayer spacing relative to all the other membranes, thereby promoting rapid solvent and low-MW solute transport by reducing steric hindrance and increasing the number of accessible transport paths. The rSG-GO membrane exhibited markedly higher rejection of low-MW species, with slightly reduced permeance compared to rTBO-GO. This enhanced selectivity is attributed to the more compact interlayer spaces formed by the SG intercalants.

**Figure 3:**
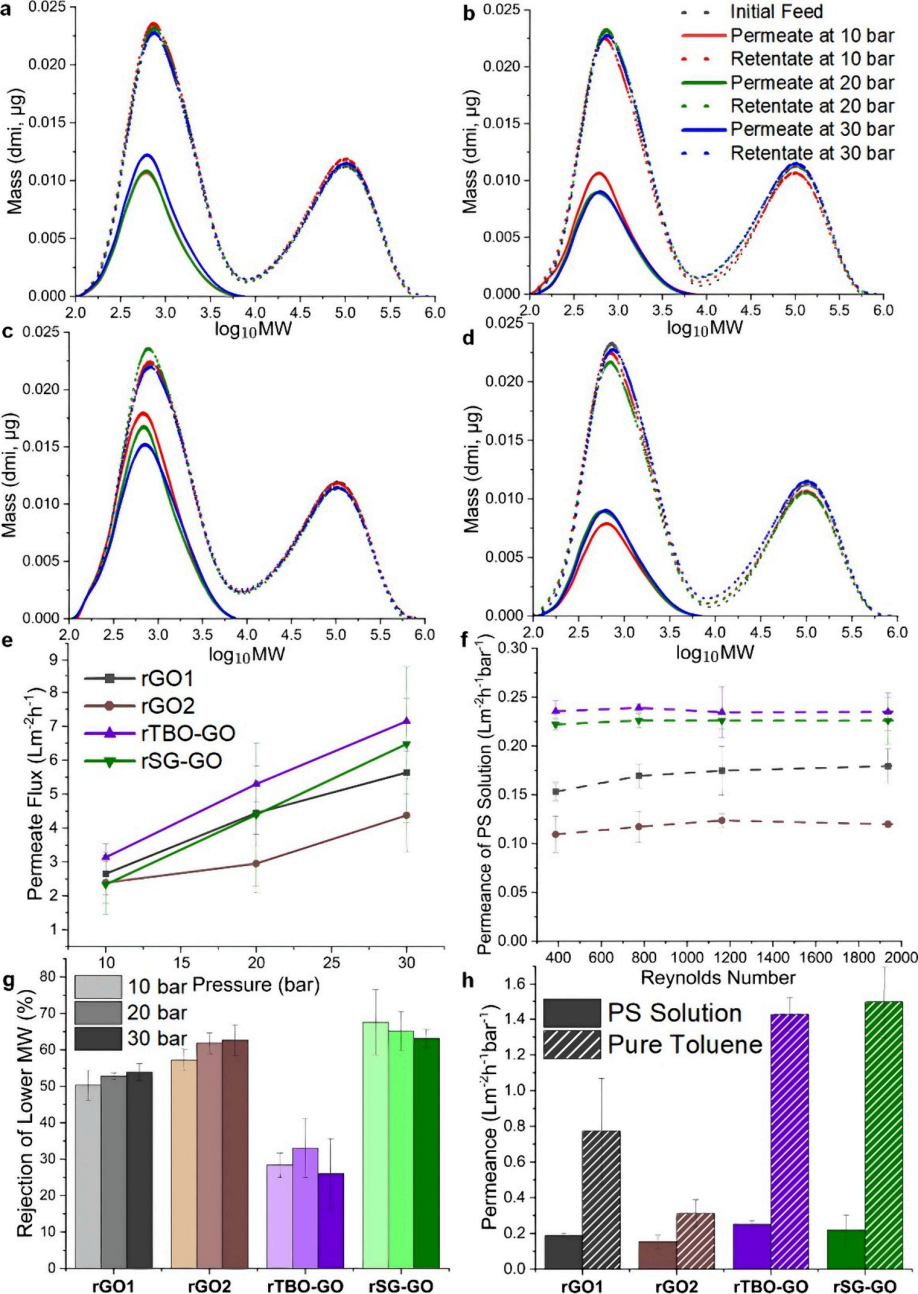
Molecular weight distributions of poly­(styrene) (PS) in permeates and retentates at varying transmembrane pressures for (a) rGO1, (b) rGO2, (c) rTBO-GO, and (d) rSG-GO membranes at 25 °C. (e) Permeate flux dependence on pressure for all membranes. (f) Permeance vs average crossflow Reynolds number for all membranes at 30 bar. (g) Rejections of low-MW species (log_10_MW 2–4); bar shading encodes pressure: light = 10 bar, medium = 20 bar, dark = 30 bar (applies to all membranes). (h) Permeance comparison in PS solution (solid) and pure toluene (hatched); values are the slope of J vs ΔP plots over 10–30 bar.

X-ray diffraction was used to track the average interlayer *d*-spacings of nonreduced GO, TBO-GO, and SG-GO (Figure S4b–e). These serve as structural proxies, since reduced membranes have much lower long-range order resulting in very broad/undetectable peaks. The dry GO membrane exhibited average interlayer spacing ∼7.5 Å, which swelled to ∼13 Å in water/ethanol (polar solvent intercalation), whereas there was negligible swelling in toluene or PS solution. Pillaring altered the gallery size and stability: the dry TBO-GO and SG-GO membranes showed average interlayer spacings ∼12 and ∼8 Å, respectively. Importantly, both maintained these *d*-spacings in polar and nonpolar solvents as well as PS solution, indicating stabilized galleries consistent. Although the SG-GO membrane exhibits a more compact average (XRD) interlayer spacing the nonpillared GO membranes, the reduced rSG-GO membrane has higher interlayer spacing than the reduced rGO (nonpillared) membrane, as corroborated by the estimated effective pore size distributions (Figure S4e,f). Pillaring stabilizes the nanosheets and suppresses gallery collapse, i.e., reduces the number of constrictions/bottlenecks, upon reduction.^14^ Figure 3f shows that the permeance is independent of the Reynolds number (*Re*), which was varied by changing the crossflow feed velocity. At the same time, Figure 3h shows that the permeances during nanofiltration of the PS feedstock decrease by a factor of 3–7× relative to the permeances of pure toluene. Taken together, the two findings indicate that the permeance decreases during nanofiltration are less likely to be caused by external mass transfer resistances (such as concentration polarization), but rather due to the stronger adsorption of PS oligomers in the interlayer spaces, leading to slower solvent (toluene) transport. Solute adsorption/binding on the membrane surface as well as in the nanochannels—in this case, oligomer adsorption—is a well-established contributor to solvent permeability and solute rejection nanofiltration.^37–39^ The reported membrane permeances include the combined effect of the ∼130 nm rGO coating, the PVDF (nominal 30 nm pore size) support that is available industrially at roll scale, and the cake/concentration polarization resistance of large solutes on the membrane surface. After hydraulic compaction (which occurs naturally during operation), the bare PVDF support permeance (Figure S5) is substantial but expectedly lower than typical large-pore supports (e.g., 200 nm pore size nylon Whatman filters).^40^ To demonstrate facile enhancement of permeance, we fabricated a thinner rTBO-GO membrane (∼40 nm) according to our previously reported methods.^14,41^ This increased the pure toluene permeance by ∼8×, and increased the permeance by ∼2× in the PS solution (Figure S6a) while preserving complete high-MW retention (Figure S6b). Thus, while this study emphasizes the key issues of sharp MW fractionation and stability for process relevance, higher throughput is easily accessible by membrane thinning and optimization/selection of industrially available supports.

Notably, all the membranes achieved complete retention of high-MW species (log_10_MW > 4), confirming the robustness of the GO-based selective membrane layer in maintaining size-exclusion nanofiltration. The rTBO-GO, with its relatively lower rejection of low-MW oligomers and higher flux, is more suitable for the selective removal of low-MW components, allowing the high-MW retentate fraction to be efficiently recycled to the depolymerization reactor. In contrast, rSG-GO could be better suited for applications requiring tighter MW cutoffs, such as the further fractionation of the low-MW oligomer permeate for different uses such as refinery aromatics blendstock, lubricants, resins, adhesives, and additives.^42–46^ Together, these results demonstrate that rGO-based OSN membrane performance can be tuned through the choice of pillaring agent and degree of reduction, enabling different separation objectives in polymer recycling processes. The poly­(vinylidene fluoride) PVDF substrate (with no rGO membrane layer) was also subjected to permeation measurements and exhibited no discernible separation (Figure S3), confirming that molecular separation was governed entirely by the GO-based selective layer.

Extended OSN nanofiltration measurements were conducted to assess the longer-term performance of the rTBO-GO membrane (Figure 4). The 10 wt % poly­(styrene) (PS) solution in toluene was processed continuously for over 600 h (∼25 d) in a crossflow system (30 bar, 0.57 L/min), with toluene replenished periodically to match permeate withdrawal, i.e., operation in diafiltration mode (see Experimental Methods). GPC molecular weight profiles (Figure 4a) revealed a steady increase in low-MW components in the permeate, while high-MW fractions were consistently retained in the retentate. The high-MW fraction peak in the GPC spectrum remained unchanged over time. Minor shifts observed in the low-MW range may result from some mechanical degradation during prolonged shear exposure, a known effect in polymer solutions.^5,47^ The membrane exhibited stable operation with no observable fouling or flux decline, yielding an average flux of 8.1 ± 0.8 L m^–2^ h^–1^ (Figure 4b). The cumulative “cut” of the low-MW fraction in the permeate stream (Figure 4c) was continuously calculated (eq S15), and its value reached unity after ∼600 h, denoting complete separation of the low-MW fraction from the retentate (Figure 4c). The rTBO-GO membrane showed no sign of visible peeling, fouling, or degradation after 600 h of OSN operation (Figure S1, bottom panel). Further postoperation characterization corroborates long-term stability. After >600 h of PS solution nanofiltration, XPS (Figure S7) shows nearly identical spectra relative to the pristine membrane. SEM (surface and cross-section, Figure S8) reveals no delamination, peeling or cracking. Together with the stable flux (Figure 4b), these results indicate excellent chemical and morphological integrity of the rTBO-GO membrane over extended operation.

**Figure 4:**
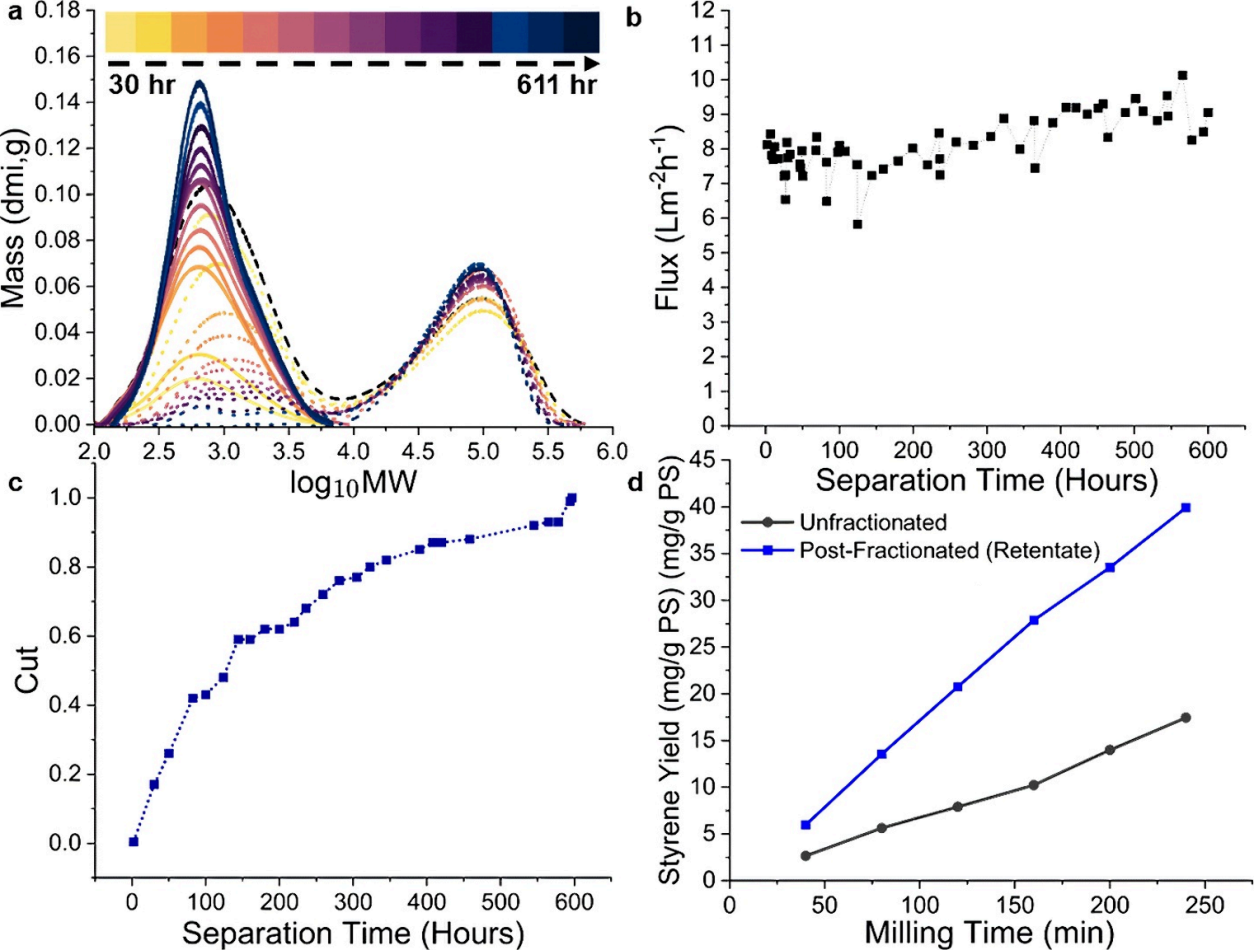
(a) Time-resolved MW distributions of retentate and permeate over 611 h of operation: initial feed (black dashes), permeate (solid), and retentate (dotted). (b) Permeate flux stability over time. (c) Permeate cut progression showing low-MW fraction recovery. (d) Styrene yield from mechanocatalytic recycling of PS for unfractionated and postfractionated PS solutions.

As mentioned earlier (Figure 1), MW fractionation is expected to have a significantly positive effect on depolymerization efficiency. To evaluate this effect, the high-MW retentate stream obtained after >600 h of fractionation was subjected to mechanocatalytic ball milling^33^ after evaporative solvent removal (see Experimental Methods). Compared to the unfractionated PS pellets, the high-MW fraction (obtained from OSN with the rTBO-GO membrane) yielded approximately twice the amount of styrene monomer (Figure 4d). This enhancement in yield can be attributed to both thermodynamic and kinetic factors.^48,49^ Conversion of PS to its monomer requires at least two elementary steps: chain scission, followed by depropagation.^50^ Mechanocatalytic depolymerization proceeds more effectively when the feedstock comprises high-MW, unbranched polymer chains, which undergo chain scission more readily and predictably under mechanical force compared to low MW oligomers, which are unreactive toward depolymerization and are instead more prone to acting as chain transfer agents that can interfere with the depropagation reaction.^51^ The presence of low-MW oligomers in the feedstock introduces volatility, poor energy transfer during milling, and side reactions that reduce the efficiency and selectivity of depolymerization. Removing these low-MW species prior to depolymerization improves heat and momentum transfer during milling, increases catalyst-polymer chain contact, and reduces mass transport heterogeneity. Thermodynamically, the removal of low-MW species shifts the reaction equilibrium further toward monomer production by decreasing the initial concentration of nonreactive fragments. OSN with hydrocarbon-stable rGO membranes offers efficient fractionation and lower energy consumption by operating at ambient temperatures and avoiding phase changes. Additionally, toluene from both retentate and permeate streams is readily recovered via simple vacuum distillation, enabling solvent reuse.^8^

In summary, chemical reduction combined with π-conjugated molecular pillaring provides a controllable route to tune GO interlayer galleries for OSN in nonpolar solvents. Among the variants, rTBO-GO delivers the best process balance of the stable and high flux with sharp retention of high-MW PS and selective passage of low-MW oligomers (log_10_MW ≈ 2–4), and maintains performance over >600 h. rSG-GO affords a slightly tighter cutoff at modestly lower permeance, whereas over-reduction (rGO2) increases selectivity at the expense of flux. Functionally, coupling rTBO-GO fractionation to mechanocatalytic depolymerization nearly doubles styrene yield relative to unfractionated PS, consistent with removal of oligomers that decrease energy transfer and hinder chain-end depropagation. The retentate enriches high-MW chains for efficient depolymerization or reuse. Additionally, the permeate provides a styrene oligomer stream suitable for valorization by multiple routes. These results demonstrate that tunable intercalated rGO laminate membranes enable precise molecular-weight control in hydrocarbon media and offer a practical separation handle to upgrade recycling efficiency.

## Supplementary Material



## Data Availability

Additional data/scripts related to this paper may be requested from the authors.

## Notes

All details of experimental procedures, including materials used, membrane fabrication methods, stream characterization techniques, nanofiltration measurement and analysis methods, and mechanochemical depolymerization conditions; relevant literature methods; interlayer modification, reduction conditions, and C/O ratios of GO-based membranes; numerical rejection of low-MW fraction values; permeance in PS solution and pure toluene; membrane appearance before and after PS fractionation; crossflow filtration setup; MW distribution profiles; additional XPS, XRD, MWCO, and pore size distribution data; bare PVDF support permeance vs solvent viscosity plot; and SEM images of rTBO-GO membrane (PDF)
