# Supplementary material for: Graphene Oxide Membranes for Sustainable Recycling: Poly(styrene) Fractionation by Organic Solvent Nanofiltration
Source: ACS Eng Au. 2025 Dec 31;6(1):82–9. doi: 10.1021/acsengineeringau.5c00102 (PMC12921688; doi:10.1021/acsengineeringau.5c00102)
Supplement: Supplementary file 1 [file eg5c00102_si_001.pdf]

Supporting Information

for

Graphene Oxide Membranes for Sustainable Recycling: Poly(styrene) Fractionation by Organic Solvent Nanofiltration

Natechanok Yutthasaksunthorn[1], Yuchen Chang[1], Van Son Nguyen[1], Kaung Su Khin Zaw[1,2], Scott A. Sinquefield[2], Carsten Sievers[1,2], Sankar Nair[1,2*]

[1]*School of Chemical & Biomolecular Engineering, Georgia Institute of Technology, Atlanta, GA, 30332, USA*

[2]*Renewable Bioproducts Institute, Georgia Institute of Technology, Atlanta, GA, 30332, USA*

* Corresponding author: sankar.nair@chbe.gatech.edu

Number of pages: 27

Number of supplementary tables: 3

Number of supplementary figures: 8

Experimental Methods

Materials and Chemicals

Sulfuric acid (98% H_2SO_4), graphite powder (synthetic grade, lateral size hydrochloric acid (HCl), sodium hydroxide (NaOH), potassium persulfate ($K_2S_2O_8$), phosphorus pentoxide (P_2O_5), potassium permanganate ($KMnO_4$), and pH 13 buffer were purchased from SigmaAldrich (Milwaukee, MI). A Thermo Scientific 7128 RO system produced deionized (DI) water. 30 nm pore size Polyvinylidene fluoride (PVDF) substrate was obtained from Sterlitech Corp. Using DI water (conductivity = 4.5 µS, 25 °C) was supplied by the building. Pure ethyl alcohol and reagent grade toluene (99.5%) for organic solvent nanofiltration measurement were purchased from Sigma-Aldrich (Milwaukee, MI). Powder Sodium Sulfate (Na_2SO_4, ≥99.0%, anhydrous), Hydriodic acid, 57 wt. % in H_2O for membrane reduction was purchased from SigmaAldrich (Milwaukee, MI)

Polystyrene Feedstock and Mechanocatalytic Recycling Process

The polystyrene (PS) pellet used in this study was purchased from Sigma-Aldrich (Product No. 331651), a general-purpose, bimodal molecular weight PS. This commercial PS feedstock was selected due to its representative molecular heterogeneity, making it suitable for evaluating membrane fractionation and subsequent recycling performance.

For separation experiments, the PS pellet was dissolved in toluene at 10 wt% to ensure consistent feed composition and supply throughout long-term nanofiltration. This solution reflects similarity to PS composition subjected to mechanocatalytic depolymerization, allowing direct comparison between untreated and fractionated streams.

Mechanochemical depolymerization was conducted following the protocol from Chang *et al.*(Chang et al., 2024a) on a Retsch MM400 ball mill, using stainless steel jars and balls. In our

study, ~1 g of fractionated or unfractionated PS was milled at 30 Hz in a 25 mL jar with eight 10 mm diameter balls to produce solid residues and volatile products for subsequent GPC and GC-MS analysis. Styrene monomer yield was quantified using gas chromatography to assess the efficiency of depolymerization, according to previously reported procedures (Chang et al., 2024a).

The membrane-separated retentate, enriched in high-MW fractions, was subjected to this mechanochemical depolymerization process. Comparison with the unfractionated PS feedstock showed significantly enhanced monomer recovery from the retentate, affirming the benefit of membrane fractionation in removing low-MW inhibitors and improving reactivity of the feedstock.

GO Synthesis

GO was synthesized by a modified Hummers' method The detailed methods used for determining these characteristics are described in our prior work (Supporting Ref 1). The prepared GO suspension was used for membrane fabrication.

Membrane Fabrication

All membranes were prepared by a pressure-assisted filtration method onto PVDF substrates (47-mm-diameter Millipore filters with 0.2-μm pore size). To obtain uniform and homogeneous membranes, the stock GO suspension was diluted to a concentration of 1 mg ml^{-1} before membrane preparation. To fabricate intercalated/pillared GO membranes, we used our prior method, detailed in (Wang et al., 2021) for pillaring TBO-GO membranes and the method of (Supporting Refs 2-3) for pillaring SG-GO membranes. TBO- and SG-intercalated GO membranes were prepared by mixing the pillaring materials and GO suspension at a 1:1 weight ratio. The resulting membranes exhibited final weight percent loadings of 27 wt% for TBO-GO

and 32 wt% for SG-GO, as calculated from **Eq. S1**. Since the vacuum filtration is carried out in a 'dead-end' mode, the degree of pillaring incorporation in the membrane is determined from the difference in pillaring concentration between the initial solution and the vacuum filtrate after membrane preparation (as measured by UV-vis spectrophotometry). After the vacuum filtration, the permeate was collected and its residual concentration was determined with a Cary-60 UV-vis spectrophotometer (Agilent Technologies). The pillaring loading content in the membrane can then be determined by the following formula:

$$\text{Pillaring Molecule Loading (wt.\%)} = \frac{c_B V_B - c_P V_P}{m_{GO} + m_{Pillaring}} \times 100\% \qquad \text{(Eq S1)}$$

Where c_B is the pillaring concentration in reagent B, V_B the volume of reagent B used in the preparation of the vacuum filtration solution, c_P the vacuum filtration permeate side TBO concentration measured by UV-vis, c_P the volume of the permeate, m_{GO} the mass of the GO deposited on the membrane surface (assumed identical to the mass of GO in the suspension) and $m_{Pillaring}$ the mass of the pillaring deposited on the membrane surface.

Reduction of the membranes was performed using hydriodic acid (HI) as reductant. HI is chosen for the tunable reduction process because: (1) it is a strong enough reducing agent that can operate at room temperature, and (2) compared to other commonly used reducing agents, such as hydrazine or sodium borohydride, it is generally considered to be a more cost-effective option and feasible to be scaled up. Initially, HI-vapor reduction processes at 80°C were utilized to reduce GO membranes. However, the vapor reduction method exhibited difficulties in reproducibly controlling the membrane composition. Therefore, a modified HI reduction process was implemented (Yutthasaksunthorn et al., 2025) . A vacuum-assisted direct reduction method, conducted at room temperature, enabled successful control of the reduction degree and quality of the fabricated reduced graphene oxide (rGO) membranes with a range of

hydrophobicity/hydrophilicity by reducing different fractions of hydrophilic group on the GO membrane layers. In this process, lower concentrations of the HI reductant (5.7 wt% aqueous), could be used. This method is also more scalable and amenable to use on a larger scale in the future.

Separation Procedure

The crossflow cell setup, illustrated in **Figure S2**, was employed for all separation experiments. The polystyrene (PS) solution separations were conducted at room temperature under a fume hood, with an average operating temperature of 22.5°C (ranging between 20°C and 23.5°C) over the 30-day experimental period for the long-term fractionation.

Crossflow Nanofiltration and Diafiltration

Continuous nanofiltration and diafiltration were carried out in a Sterlitech CF047 stainless-steel crossflow cell (effective circular area Ø ≈ 4.7 cm; channel height ≈ 0.19 cm). Using the manufacturer's channel geometry, this corresponds to a nominal cross-channel velocity of ≈7.1 cms^{-1}; Reynolds numbers were computed using the solvent properties as shown in **Eq S8** **(**confirming laminar flow under all conditions). Transmembrane pressure (TMP) was controlled at 10–30 bar unless stated otherwise. All contacted parts (cell body, tubing, fittings, gaskets) were 316 SS or PTFE to ensure compatibility with toluene and PS solution. During long-term operation, the system was operated continuously at the target TMP with periodic sampling (feed, permeate, retentate) every 2-24 hours. Flux was recorded from permeate mass on a gravimetric balance. For diafiltration experiments, toluene was metered into the feed tank at a rate equal to the permeate outflow while holding Diafiltration, thereby maintaining constant volume and steady hydrodynamics as solute concentration decreased.

Pressure-Dependent Separation

Pressure-dependent separation experiments were conducted at a constant flow rate of 190 mL/min (equal to a Reynolds number Re = 387). Membrane performance was evaluated under sequentially applied transmembrane pressures of 10, 20, and 30 bar. Permeate flux and solute rejection were monitored at each pressure level to assess the influence of pressure on the separation efficiency and membrane selectivity during polystyrene (PS) fractionation.

Flowrate-Dependent Separation

Flowrate-dependent separation experiments were conducted at a constant transmembrane pressure of 10 bar, with additional measurements performed at both 10 and 30 bar for specific sample for running long term. To verify the absence of mass transfer limitations, flow rate variations (190 – 950 mL/min (Re between 387-1937) were applied while monitoring solute rejection (R) and permeance (P). Rejection ($R = 1 - \frac{C_p}{C_f}$) was determined from the permeate (C_p)and feed (C_f) concentrations, while permeance ($P = \frac{J}{\Delta P}$) was calculated from the flux (J) and applied pressure (ΔP). Stable rejection and permeance across varying flow rates indicated that solute transport was governed by membrane selectivity rather than external hydrodynamic effects. Reynolds number for each flow rate was calculated using the equation of $Re = \frac{\rho u d_h}{\mu}$ as exhibited in the following section. Based on the calculated values of Re, the flow in the membrane channel was determined to be laminar, as all values were below the threshold of Re < 2000. This laminar flow regime ensures consistent and predictable transport properties within the system, minimizing the impact of turbulence on the separation performance of the membrane (detailed calculation in supplementary information**.** This experimental design ensured systematic

evaluation of membrane performance under varying pressure and flowrate conditions, providing insights into the membrane's separation efficiency and operational stability.

Solvent Recovery by Distillation

2 L of polymer/toluene solution and an egg-shaped PTFE stir bar were charged to a 4 L round-bottom flask fitted with two ≈60 cm fractionation columns in series, a water-cooled condenser, and a 2 L round-bottom receiver. Thermometers were placed in the boiling flask and at the top of the second column to monitor temperatures. The boiling flask sat on a hemispherical contact heater with a temperature controller, placed atop a magnetic stirrer; stirring was engaged throughout. Heating was adjusted so that the vapor temperature at the column head matched the ambient boiling point of toluene (local atmospheric pressure). Distillation continued until condensate transfer into the receiver ceased. The recovered toluene was reused when it met identity/purity checks (any two of: density 0.866–0.868 g mL^{-1} at 20 °C, refractive index 1.496–1.497 at 20 °C, GC-FID ≤0.5 area% non-toluene peaks, or non-volatile residue ≤200 ppm after evaporation). The viscous polymer residue remaining in the boiling flask was poured into an evaporating dish and allowed to dry in a fume hood until (i) the material hardened (high-MW fraction) or (ii) no toluene odor was evident (low-MW fraction). The high-MW fraction obtained by this procedure was subsequently recycled as feed for mechanochemical depolymerization experiments.

Characterizations

X-ray diffraction (XRD) measurements were performed using a Rigaku MiniFlex600 diffractometer equipped with Cu Kα radiation (λ = 1.5406 Å) in the 2θ range of 3° to 50°. The measurements were carried out with a step size of 0.01° and a scanning speed of 5°/min to ensure high-resolution peak detection. Due to the weak X-ray intensity observed for membranes

fabricated from a 1 mg/L GO dispersion, membranes prepared with a 10 mg/L dispersion were used to amplify the signal for analysis.

To investigate the influence of different organic solvents (water, ethanol, hexane, toluene) and a 10 wt% polystyrene (PS) solution in toluene, both pillared and non-pillared GO membranes were tested. Prior to XRD analysis, the membranes were subjected to the following treatment:

1. Vacuum Drying: Membranes were kept under vacuum for over 48 hours to remove interlayer water.
2. Solvent Immersion: Membranes were immersed in the selected solvents for over 3 days and for 2 weeks in the 10 wt% PS solution in toluene.
3. Drying Before Measurement: To avoid solvent diffraction effects, the membranes were removed from the solvents and air-dried for 2–5 minutes immediately before measurements.

This systematic procedure allowed for accurate characterization of the interlayer spacing (d-spacing) changes in both pillared and non-pillared membranes, providing insights into the structural response of the GO-based membranes under diverse solvent conditions.

X-ray Photoelectron Spectroscopy (XPS) measurements were performed using a Thermo K-Alpha XPS system equipped with a monochromatic Aluminum Kα (Al Kα) source at 1.486 keV. The technique enables the detection of elements from Li (Z = 3) to U (Z = 92), providing surface composition, elemental abundance, and chemical bonding states with a detection depth of approximately 5 nm. To ensure consistent and reliable results, the following sample preparation protocol was used:

1. Vacuum Drying: All membranes were vacuum-dried for 48 hours prior to measurement to minimize pumping time and eliminate any residual water, which could interfere with surface chemistry.
2. Membrane Samples: The same membranes used for permeation experiments were analyzed to monitor chemical stability and changes.
3. Measurement Conditions: XPS analysis was performed before and after membrane reduction (both pillared and non-pillared variants) and before and after exposure to various organic solvents and a 10 wt% polystyrene solution in toluene.

The goal of the analysis was to determine surface elemental composition and detect any chemical changes induced during the reduction process or after the separation experiments. This approach enabled evaluation of the surface chemical stability of the membranes under varying operational conditions, providing insights into their performance and robustness for organic solvent nanofiltration.

Permeation Measurements

To quantify the nanofiltration (NF) properties of the membranes, permeation measurements were conducted with pure water, organic solvents (ethanol and toluene in this study), and PS solution. Two sets of experiments are performed to study the reduction degree effect and the combination of intercalation and reduction effect at different transmembrane pressures (TMPs) at room temperature. The flux (J) at each TMP was calculated using Equation (S2):

$$\text{Flux} \quad (J) = \frac{\Delta m}{A\,\rho \Delta t} \qquad \text{(Eq S2)}$$

whereΔ m is the mass of the permeate collected at each time (Δt), A is the effective area of the membrane, and ρ is the density of the permeate.

For all separation measurements, the rejection (R) can be calculated using Equation (3):

$$\text{Rejection } (R) = 1 - (\frac{C_p}{C_f}) \quad \text{(Eq S3)}$$

where C_p and C_f are the permeate and feed sample concentrations.

Viscous flow through the membranes as governed by the Hagen-Poiseuille law, describes the relationship between permeate flux and several parameters as demonstrated by the following equation:

$$J = \frac{\varepsilon \pi r_p}{8\mu\delta\tau}\Delta p \quad \text{(Eq S4)}$$

Where J is the flux of water and organic solvents ($m^3m^{-2}s^{-1}$), Δp is the transmembrane pressure (Pa), ε is the surface porosity, r_p is the average pore radius (m), μ is the liquid viscosity (Pa·s), δ is the membrane thickness (m) and τ is the membrane tortuosity

The Hagen-Poiseuille equation can be modified into Eq. (S5) for 2D lamellar membranes

$$Flux = \frac{h^4\Delta p}{12L^2h\Delta x} \quad \text{(Eq S5)}$$

where h is the d-spacing between GO nanosheets, Δp is the transmembrane pressure, L is the modal lateral dimension of the GO nanosheets, η is the viscosity of the solvent and Δx is the thickness of the GO membrane.

Crossflow Velocity Calculation

$$Crossflow\ Velocity\ (cm/s) = \frac{Volumetric\ flowrate\ (cm^3/s)}{Sectional\ Area\ (cm^2)} \quad \text{(Eq S6)}$$

$$Sectional\ Area\ (cm^2) = Half\ Diameter\ (cm)\ \times Channel\ Height\ (cm) \quad \text{(Eq S7)}$$

Reynolds Number Calculation

$$Re = \frac{\rho u d_h}{\mu} \quad \text{(Eq S8)}$$

where ρ = Fluid density (kg/m³), u = Crossflow velocity (m/s), d_h = Hydraulic diameter (m), and μ = Dynamic viscosity of the fluid (Pa·s).

The hydraulic diameter is defined as:

$$d_h = \frac{2WH}{W+H} \quad \text{(Eq S9)}$$

where W = Width of the channel (m), H = Height of the channel (m)

The Reynolds number indicates whether the flow in the channel is laminar (Re<2000), transitional (2000<Re<4000), or turbulent (Re>4000). For membrane systems, laminar flow is typically observed due to low velocities and narrow channels. The Re value helps assess hydrodynamic conditions and mass transfer effects.

Example of crossflow velocity and Reynolds Number calculation

1. Crossflow cell operation for the measurements in this work ran with the flowrate of 190 mL/min which is equal to 3.17 cm^3/s

Height of crossflow cell channel (cm)	Half diameter of the effective area (cm)
0.19	2.35

The height and diameter of the crossflow cell are obtained from the manufacturer (Steritech) equipment drawing and details:

2. Sectional Area (cm^2) = 2.35 cm x 0.19 cm = 0.45 cm^2

3. $Crossflow\ Velocity\ (cm/s) = \frac{Volumetric\ flowrate\ (cm^3/s)}{Sectional\ Area\ (cm^2)} = \frac{3.17\ \text{cm3/s}}{0.45\ cm^2} = 7.06\ cm/s$

4. $d_h = \frac{2WH}{W+H} = \frac{2(2.35)(0.19)\ cm^2}{2.35+0.19\ cm} = 0.35\ cm$

5. $Re = \frac{\rho u d_h}{\mu}$

(example for toluene, the solvent used in the fractionation process in this work)

$$Re = \frac{\rho u d_h}{\mu} = \frac{\left(867\ \frac{\text{kg}}{\text{m}^3}\right)(0.0706\ m/s)(0.0035\ m)}{(0.000554\ Pas)} = 387$$

6. $Re = \frac{\rho u d_h}{\mu}$ (example for ethanol)

$$Re = \frac{\rho u d_h}{\mu} = \frac{\left(789\ \frac{\text{kg}}{\text{m}^3}\right)(0.0706\ m/s)(0.0035\ m)}{(0.00104\ Pas)} = 187$$

The Reynolds numbers are hence in the range of 187-387 (laminar flow) based upon the solvents

GPC Calibration Curve (Polystyrene Standards)

Peak edit condition (RI)

Minimum area [mV*sec]	10.000	Detection sensitivity [mV/min]	3.000
Minimum height [mV]	0.000	Base sensitivity [mV/min]	1.000
Minimum width [sec]	0.000		

Peak edit condition (UV)

Minimum area [mV*sec]	10.000	Detection sensitivity [mV/min]	3.000
Minimum height [mV]	0.000	Base sensitivity [mV/min]	1.000
Minimum width [sec]	0.000		

Calibration condition (RI)

Formula of approximation   Cubic: At3+Bt2+Ct+D

Correction   None

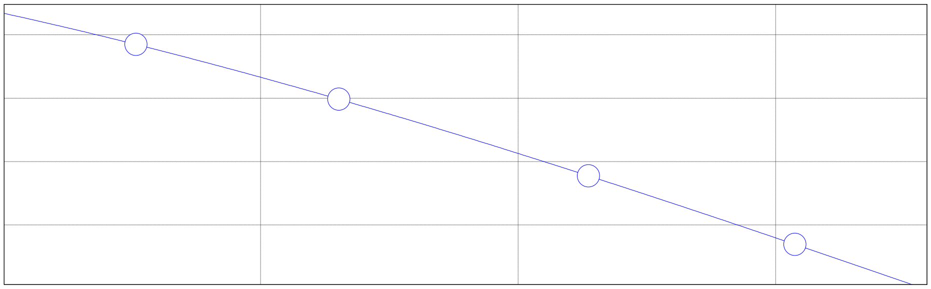

Calibration data (RI)

Time [min]	Molecular weight	Error [%]	Weight	Mark	Data name	Coefficient	
9.033	706,000	0.00000	1	STD		A =	1.273120e-003
10.608	96,400	0.00000	1	STD		B =	-6.204380e-002
12.547	5,970	0.00000	1	STD		C =	3.004318e-001
14.150	495	0.00000	1	STD		D =	7.259123e+000
						Correlation	-0.999

Calibration condition (UV)

Formula of approximation Linear: At+B

Correction Non

To accurately calculate the mass/mass fraction from the provided GPC molecular weight (MW) calibration curve and other related data, follow these detailed steps, using appropriate equations and leveraging GPC with infrared (IR) and dynamic light scattering (DLS) detection techniques:

Mass Calculation from GPC Data

Mass and molecular weight data of polymer solutions were obtained using a Tosoh EcoSEC HLC-8320GPC equipped with a TSKgel SuperMultipore HZ-M column operating at 40 °C, an internal refractive index detector (RID) and a Wyatt Technology DAWN8+ dynamic light scattering detector (DLS). The baseline eluent was chloroform containing 0.3% triethylamine at a flow rate of 0.45 mL/min.

Molecular weight distributions were obtained by matching real-time chromatogram data from the RID detector to the calibration line obtained from PS standards. Differential distributions are reported with the area under the curve normalized to 1 with respect to $\log_{10}$MW. Integral distributions are obtained as definite integrals of the corresponding differential distribution integrated up to a given value of $\log_{10}$MW, with the highest value being 1.

Calculation of total polymer mass in each sample was carried out using Wyatt Technology software interfaced with the DLS detector, which takes as input the RID detector signal from the GPC and light scattering signals from the independent DLS detector. The mass of a specific cut of the sample terminating at a given $\log_{10}$MW was calculated by multiplying the calculated total polymer mass by the value of the integral molecular weight distribution evaluated at the specified value of $\log_{10}$MW.

Step-by-Step Mass Calculation from GPC Data

1. Relationship Between Elution Time (t) and Molecular Weight (MW)

Use the calibration curve linking MW to elution time (t) to determine the molecular weight when the signal is nonzero at a specific elution time. This curve, typically plotted as log(MW vs. t, allows the determination of MW for any given retention time of the analyte.

2. Sample Injection and Dilution

Calculate the sample concentration:

$$C_{sample} = \frac{10\ \mu L}{1000\ \mu L} \times C_{original} \qquad \text{(Eq S10)}$$

where 10 μL of the sample is diluted in 1000 μL of chloroform ($CHCl_3$)

3. Use of IR Refractive Index Data

The change in the refractive index Δn is related to the sample concentration C_{sample}:

$$n - n_0 = \Delta n \propto C_{sample} \quad \text{(Eq S11)}$$

This relationship helps calculate the molecular weight distribution based on the observed calibration curve and changes in the refractive index.

4. DLS (Dynamic Light Scattering) for Absolute Molecular Weight

Use the DLS equation for molecular weight determination:

$$\frac{kC}{R} = \frac{1}{M} + 2BC \quad \text{(Eq S12)}$$

where:

- k, R, and B are constants provided by the GPC system.
- MMM represents the molecular weight as a function of concentration CCC.

5. Signal Intensity and Mass Determination

The signal intensity from the GPC data is proportional to the mass abundance of the corresponding MW fraction.

Calculate the mass differential using:

$$dW = \left(\frac{dW}{dlog(MW)}\right) \times \Delta \log{(MW)} \quad \text{(Eq S13)}$$

Here, $\frac{dW}{dlog(MW)}$ represents the mass distribution density function over the logarithmic molecular weight scale.

6. Integration for Mass Fraction

Integrate over the desired MW range to obtain the total mass fraction of interest:

$$W_{fraction} = \int_{log(MW_{min})}^{log(MW_{max})} \left(\frac{dW}{dlog(MW)}\right) d(\log(MW)) \qquad \text{(Eq S14)}$$

This integral sums the contributions of the mass fraction over the specified range of molecular weights, providing the total mass fraction of the specified MW range in the sample.

Cut Calculation Formula

$$Cut = \frac{Small\ Molecule\ (\log MW\ 2-4)\ of\ PS\ in\ permeate}{Small\ Molecule\ (\log MW\ 2-4)\ of\ PS\ in\ feed} \qquad \text{(Eq S15)}$$

Estimation of Pore Size Distributions

We selected hydrocarbon-soluble molecules spanning small aromatics to macromolecules. Each solute (20 ppm in toluene) was circulated in the cross-flow cell, then permeate and retentate concentration were quantified. Between molecules, the module was conditioned with neat toluene for ≥12 h under cross-flow condition to restore baseline. The molecule set comprised azobenzene ($C_{12}H_{10}N_2$, 182.8 g mol^{-1}), Oil Blue N ($C_{32}H_{24}N_6O_2$, 378.5 g mol^{-1}), Oil Red O ($C_{26}H_{24}N_4O$, 408.5 g mol^{-1}), fullerene C_{60} (720.6 g mol^{-1}), fullerene C_{70} (840.8 g mol^{-1}), and a polystyrene standard (PS, $M_n \approx 2000$ g mol^{-1}; repeat unit C_8H_8). The measured rejections are reported in **Figure S4e**.

Physical properties of toluene used for pore size distribution calculation:

Solvent	MW (g/mol)	dm (nm)	η (mPa s)	V_m (cm^3 mol)$^{-1}$	Density (g mL^{-1})
Toluene	92.12	0.70	0.55	106.8	0.866

Physical properties of solutes used for pore size distribution calculation:

Solute	MW (g/mol)	dm (nm)	Estimated Density (g mL^{-1})	V_m (cm^3 mol)$^{-1}$	Diffusivity in toluene (cm^2/s)
Azobenzene	182.23	0.788	1.18	154.4	1.90 x 10^{-5}
Oil Blue N	378.50	0.961	1.35	280	1.33 x 10^{-5}
Oil Red O	408.50	1.010	1.25	330	1.23 x 10^{-5}
Fullerene C-60	720.66	1.115	1.65	437	1.04 x 10^{-5}
Fullerene C-70	840.75	1.161	1.70	494	9.36 x 10^{-6}

The diameter of the solute was obtained using the following equation:

$$dm = 2\left(\frac{3V_m}{4\pi NA}\right)^{1/3} \quad \text{(Eq S16)}$$

Where V_m is the molar volume calculated from solvent density and NA is the Avogadro's number.

To correlate the molecular rejection data with pore size distribution, the rejections values of neutral solutes in toluene were used as input data into the pore flow model. The listed equations and procedure are identical to that described in **Ref. 14**, except that the rejections in the present work are obtained in toluene solvent whereas Ref 14 used aqueous-phase rejections. The Hagen-Poiseuille equation for 2D membranes such as GO membranes describes the volumetric flux (Jv) through the membrane comprising uniform nanochannels as capillaries:

$$J_{vi} = \bar{v}.4r\frac{1-\sigma}{L} \quad \text{(Eq S17)}$$

Where the porosity $\sigma = \frac{L'}{L+L'}$ (assuming L' <<< L, σ is near zero), $\bar{v}$ as the average solvent flux in pores (m/s), r as the pore size radius (nm), and L as the length of the GO sheet (nm) (L ~ 200nm

received from AFM data). The local hindrance factors, $K_{i,d}$ and $K_{i,c}$, quantify the resistance of a specific solute or ion within a pore relative to free diffusion and convection in a bulk dilute solution. The hydrodynamic interaction of solute molecules with the interlayer walls and the steric restrictions of the interlayers results from hinderances in the interlayer spaces. λ is the ratio of r_s (solute hydrodynamic radius) and r (effective pore radius). The hindrance factors are a function of λ. For transport in slit pores formed by 2D materials, the correlations for $0 < \lambda < 0.95$ are:

$$H(\lambda) = 1 + \frac{9}{16}\lambda \ln \lambda - 1.19358\,\lambda + 0.4285\,\lambda^3 - 0.3192\,\lambda^4 + 0.08428\,\lambda^5 \quad \text{(Eq S18)}$$

$$W(\lambda) = 1 - 3.02\,\lambda^2 + 5.776\,\lambda^3 - 12.3675\,\lambda^4 + 18.9775\,\lambda^5 - 15.2185\,\lambda^6 + 4.8525\,\lambda^7 \quad \text{(Eq S19)}$$

The steric partitioning coefficient $\Phi(\lambda) = (1- \lambda)$ relates to the overall hindrance factors:

$$K_{i,d} = \frac{H(\lambda)}{\Phi(\lambda)} \quad \text{(Eq S20)}$$

$$K_{i,c} = \frac{W(\lambda)}{\Phi(\lambda)} \quad \text{(Eq S21)}$$

The real/intrinsic rejection of solute (s) by the membrane can be expressed analytically:

$$R = 1 - \frac{\Phi\, \mathrm{K}_c}{1-(1-\Phi)(\exp -Pe)} \quad \text{(Eq S22)}$$

Pe is the dimensionless Peclet number for the solute :

$$Pe = \frac{K_c \bar{v}\, L}{K_s\, D_s} \quad \text{(Eq S23)}$$

Where diffusivity, D of a solute with the radius r_s is calculated using the Wilke-Chang formula:

$$D = 7.4\, \times 10^{-4}\, T \frac{\sqrt{\varphi\, MW_{sol}}}{\mu\, V_m^{0.6}} \quad \text{(Eq S24)}$$

Where MW_{sol} is the MW of solvent molecule, φ is a dimensionless solvent parameter, and V_m is the solute molar volume. Assuming R(r) is a continuous function of the pore radius, probability density factor, f (r) is used to describe the pore size distribution:

$$f(r) = \frac{1}{r\sqrt{2\pi b}} \exp - \frac{\left(\log\left(\frac{r}{r^*}\right) + \frac{b}{2}\right)^2}{2b} \qquad \text{(Eq S25)}$$

$$b = \log\left[1 + \frac{\sigma}{r^*}\right] \qquad \text{(Eq S26)}$$

To calculate the function f(r), the mean pore radius (r^*) and the standard deviation σ were calculated. The distribution function is truncated to rmax.

$$\frac{f'R(r)}{f\,R\,(r)} = \frac{1}{\int_0^{rmax} f(R)dr} \qquad \text{(Eq S27)}$$

The overall rejection over the pore radii 0 < r < rmax can be calculated using:

$$R = \frac{\int_0^{rmax} \frac{f'R\,(r) r^4\, R(r)}{\mu\,(r)\, dr}}{\int_0^{rmax} \frac{f'R\,(r) r^4}{\mu\,(r) dr}} \qquad \text{(Eq S28)}$$

Where the viscosity of the solvent is assumed constant.

Supplementary Tables

Table S1. Interlayer modification, reduction conditions, and XPS C/O ratios of GO-based membranes

Membrane Name	Pillaring Materials	Pillaring Loading (wt%)	Reduction Method	C/O Ratio ± SD	Interlayer Spacing (Å) of non-reduced membrane (before reduction)					
					Dry	Water	Ethanol	Hexane	Toluene	PS Solution
**rGO1**	None	-	HI, 5.7 wt%	11.75 ± 3.48	7.7	13.1	13.7	7.7	7.5	7.8
**rGO2**	None	-	HI, 22.8 wt%	18.50 ± 3.28	7.7	13.1	13.7	7.7	7.5	7.8
**rTBO-GO**	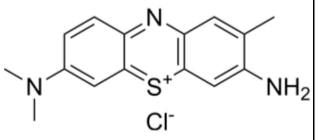 (Toluidine Blue O)	27	HI, 5.7 wt%	15.97 ± 1.66	12.2	12.5	11.7	12.1	11.8	11.7
**rSG-GO**	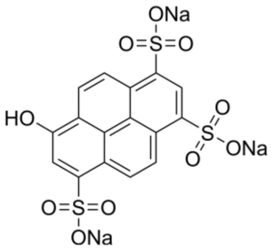 (Solvent Green 7)	32	HI, 5.7 wt%	8.32 ± 1.37	7.8	7.5	7.7	7.7	7.5	7.7

Table S2. Numerical rejection of low-MW fraction values used in **Figure 3g.** Bar labels show mean ± SD at 10, 20, 30 bar (light → dark shading). Values are mean ± SD from triplicate experiments (n=3).

Membrane Name	10 bar	SD	20 bar	SD	30 bar	SD
**rGO1**	50.28	4.13	52.78	0.94	53.9	2.35
**rGO2**	57.23	2.88	61.84	2.81	62.62	4.18
**rTBO-GO**	28.38	3.41	33.04	8.08	25.99	9.57
**rSG-GO**	67.55	8.98	65.16	5.28	63.1	2.49

Table S3. Permeance in PS solution and pure toluene at 25 °C. Values are mean ± SD from triplicate experiments (n=3).

Membrane Name	PS Solution ($Lm^{-2}h^{-1}bar^{-1}$)	SD	Pure Toluene ($Lm^{-2}h^{-1}bar^{-1}$)	SD
**rGO1**	0.19	0.01	0.77	0.29
**rGO2**	0.15	0.04	0.31	0.08
**rTBO-GO**	0.25	0.02	1.43	0.09
**rSG-GO**	0.22	0.08	1.50	0.2

Supplementary Figures

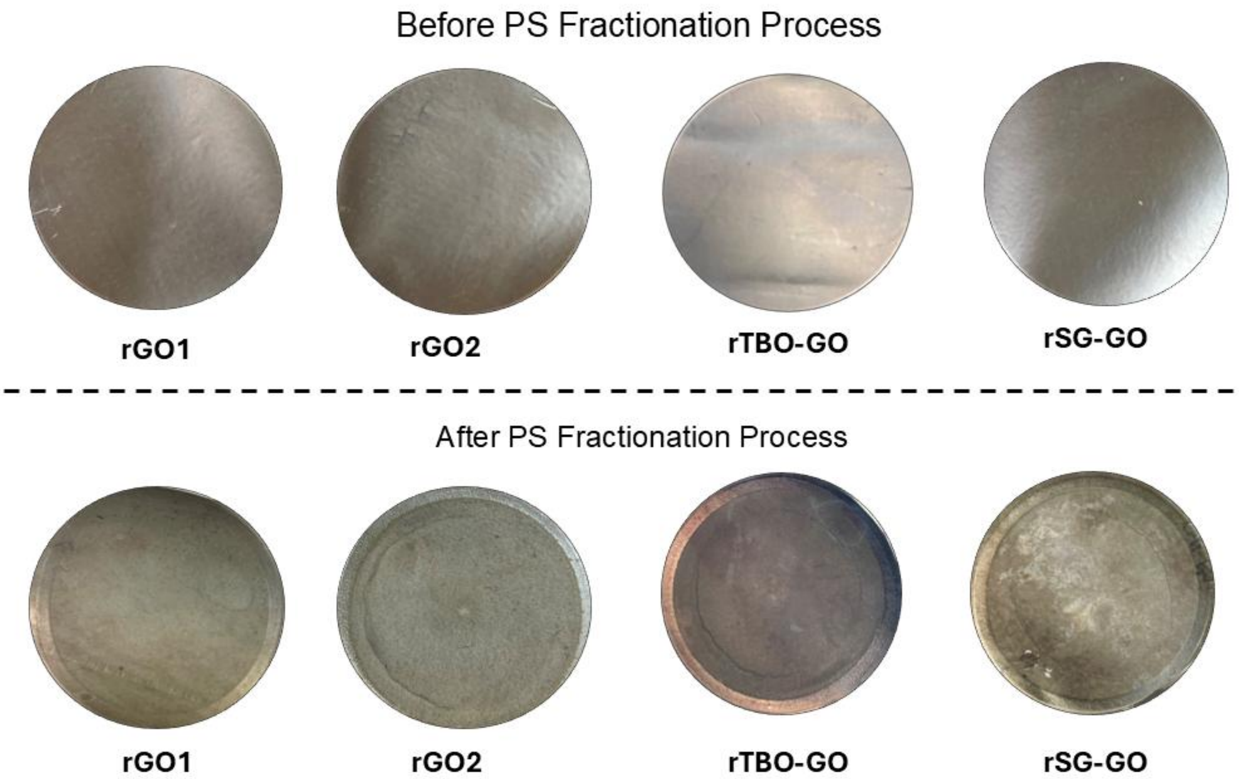

Figure S1. Membrane appearance (47 mm coupons) before and after PS fractionation using 10 wt% PS in toluene.

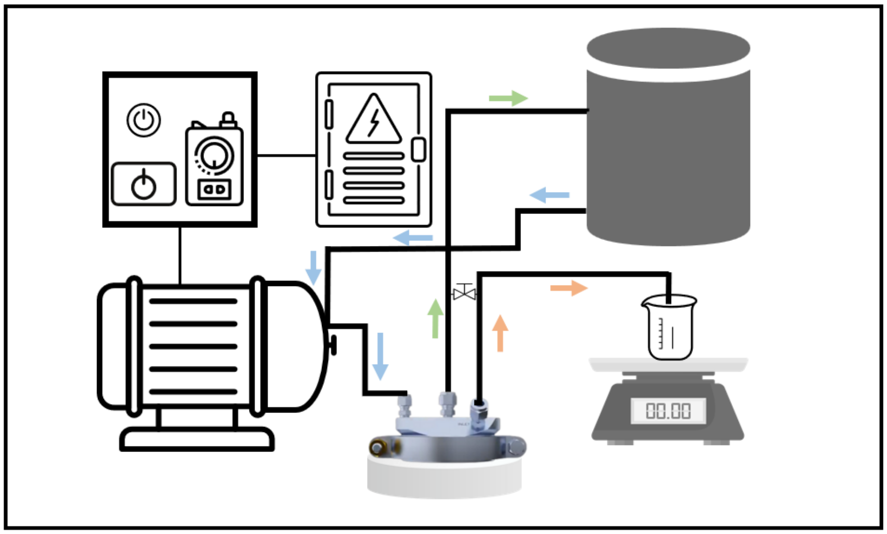

Figure S2. Schematic and photograph of the crossflow filtration setup used for membrane permeation measurements. The system consists of a power control box, variable frequency drive (VFD), and feed tank (top row), and a 3-phase motor connected to a 0.5 gal/min Catpump, CF047 membrane cell, and Ohaus 1200 g balance.

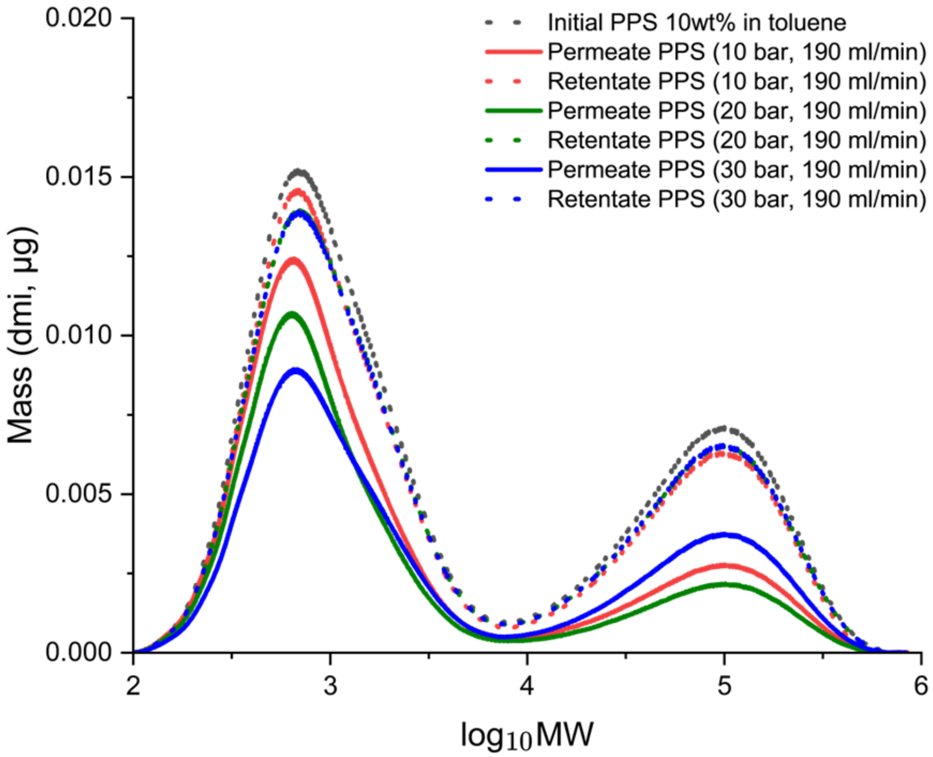

Figure S3. Molecular weight distribution profiles from PS solution filtration through bare PVDF support under various pressures, showing no separation of low- or high-MW components.

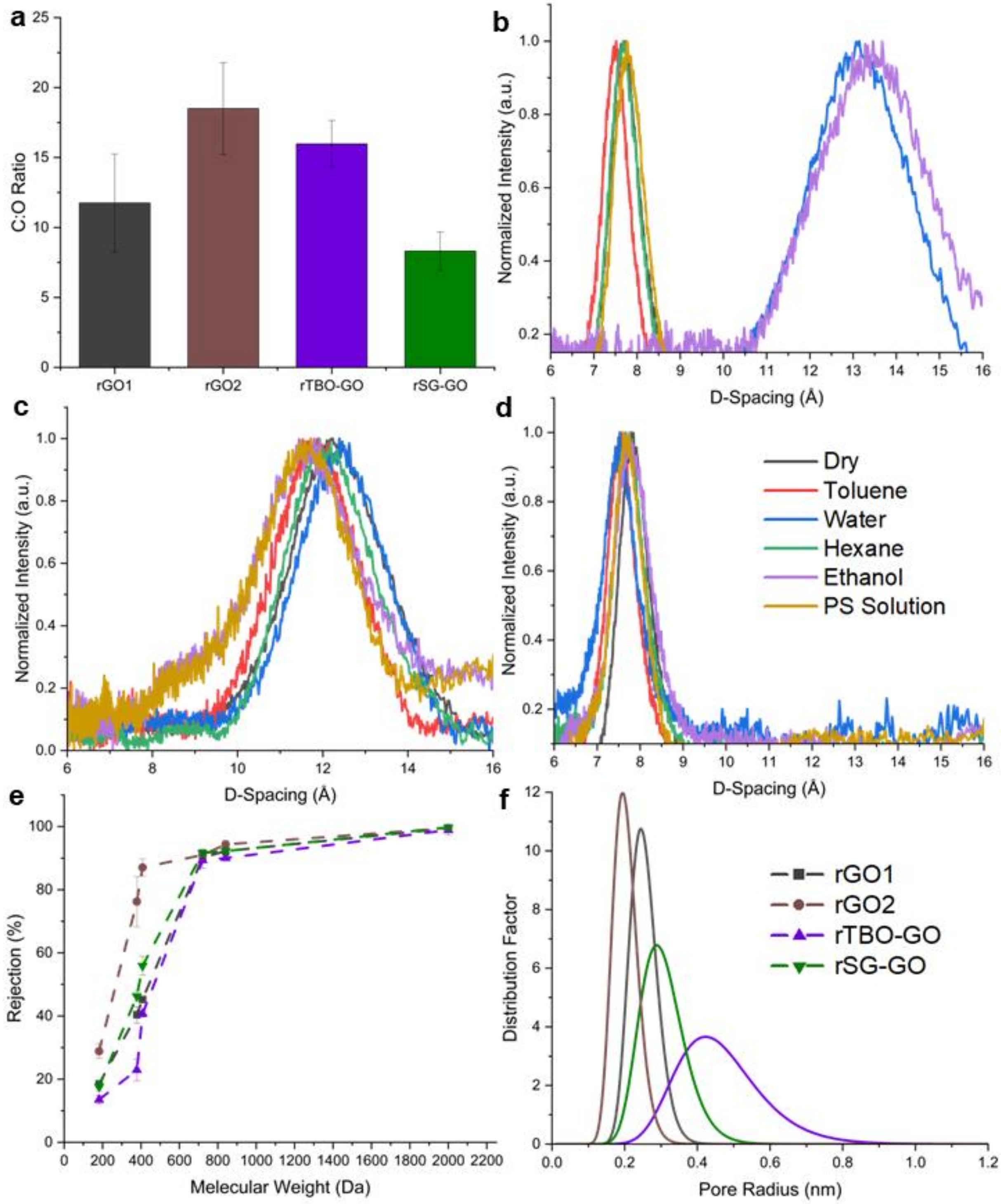

Figure S4. (a) C:O atomic ratios of membranes from XPS analysis. (b-d) Interlayer spacing profiles from XRD under various solvent environments. (e) Molecular weight cut-off (MWCO) determined from solute rejection in toluene (solute list in Supporting Information). (f) Calculated pore size distributions for each membrane.

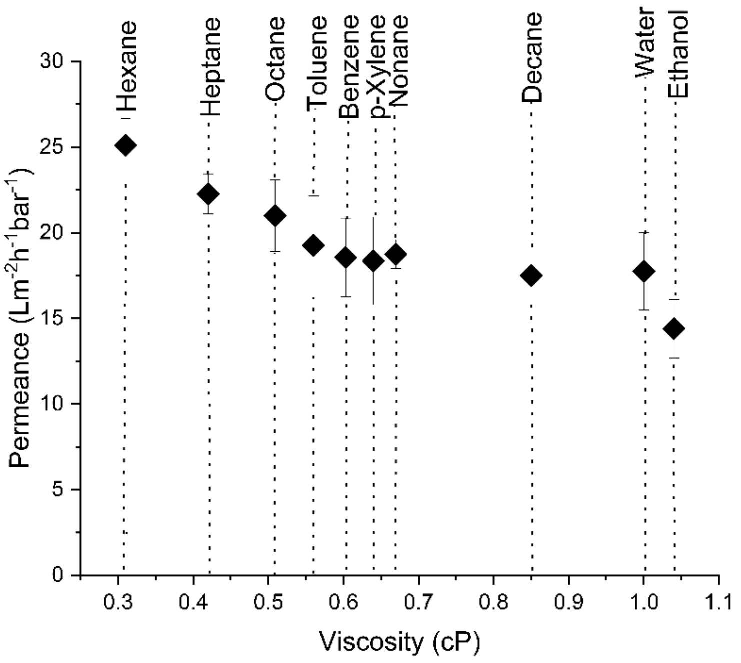

Figure S5. Bare PVDF support permeances versus solvent viscosity at 25°C.

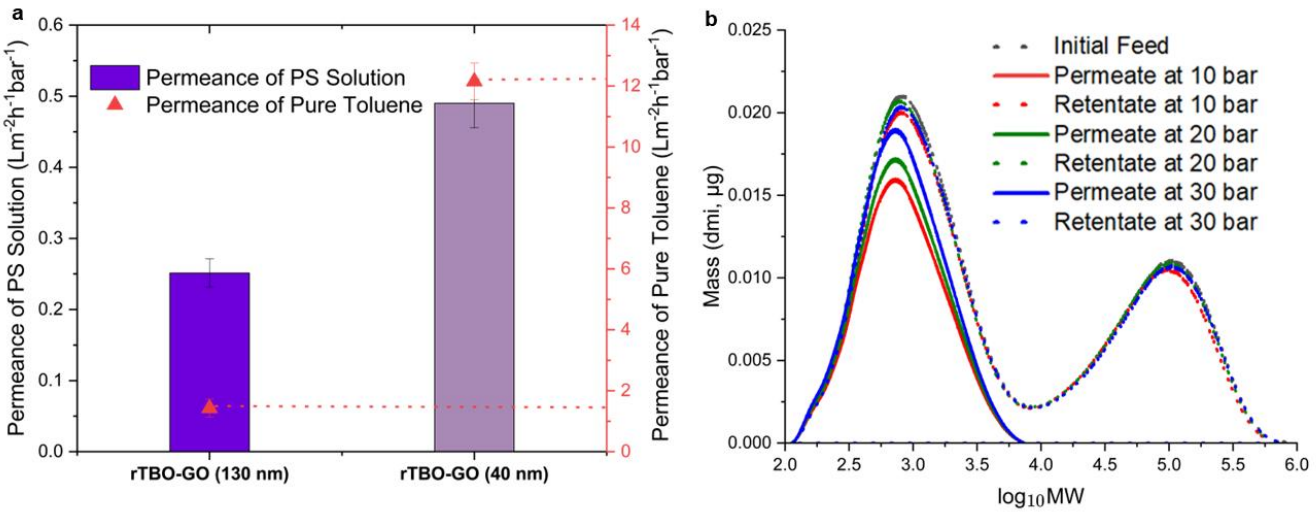

Figure S6. Effect of rTBO-GO membrane thickness. (a) Permeance in PS solution (left axis, bars) and in pure toluene (right axis, triangles), for ~130 nm vs ~40 nm thin membranes; (b) GPC traces of feed, permeate, and retentate at 10–30 bar TMP for the 40 nm rTBO-GO membrane, showing complete high-MW retention and complete permeation of the low-MW fraction.

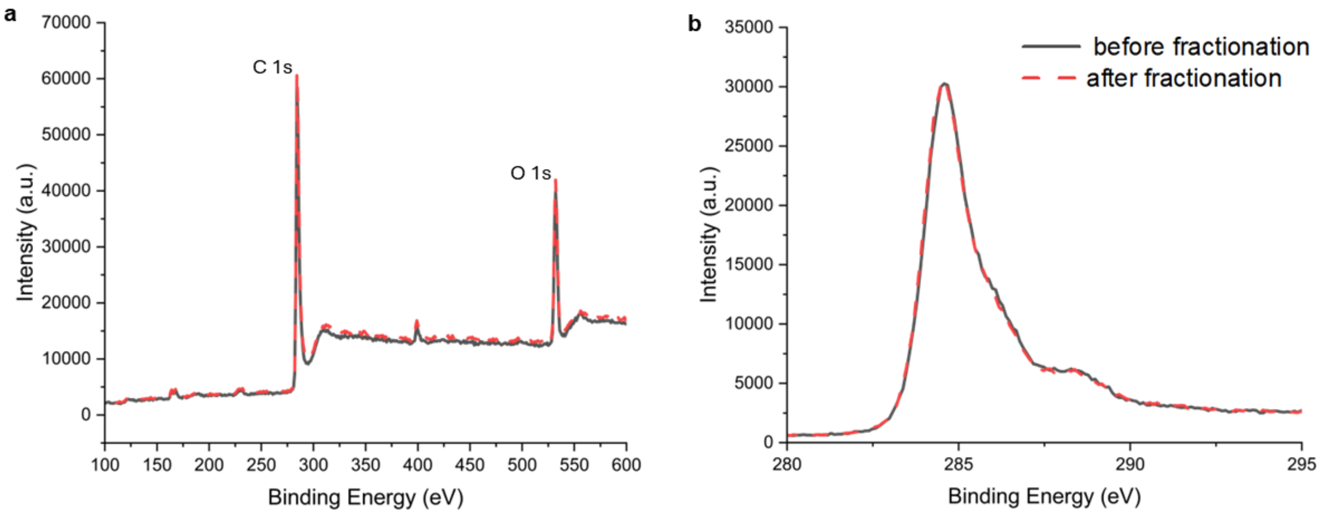

Figure S7. XPS spectra of rTBO-GO membrane before and after ~600 h PS solution fractionation: (a) survey spectra; (b) overlaid high-resolution C1s spectra.

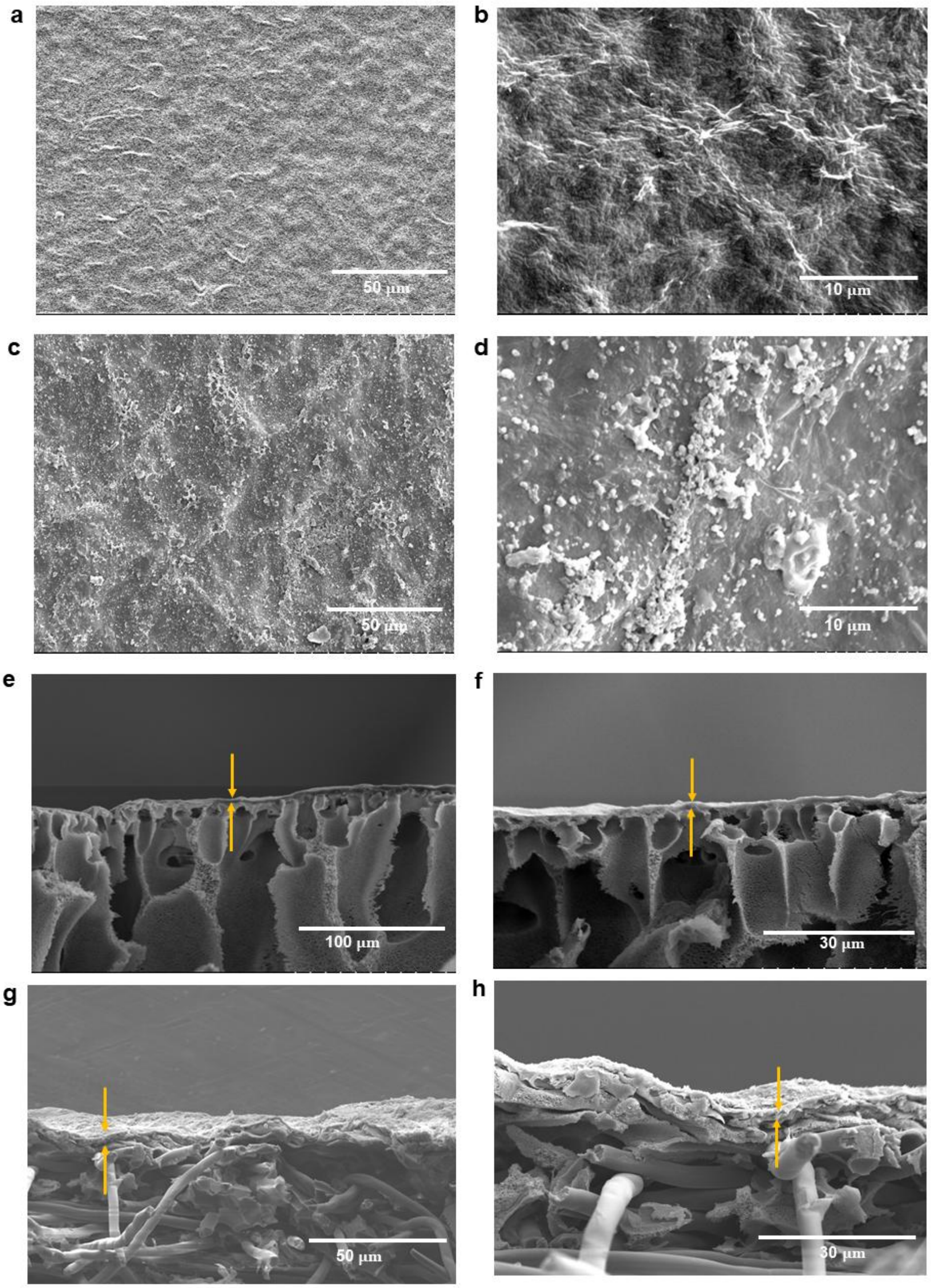

Figure S8. SEM images of rTBO-GO membrane before and after ~600 h PS solution fractionation. (a,b) Surface (top-view) before fractionation; (c,d) surface after fractionation; (e,f) cross-sections before fractionation; (g,h) cross-sections after fractionation. Yellow arrows are a guide to the eye to indicate the location of the rTBO-GO membrane on the PVDF support.

Supporting References

(1) Wang, Z.; Ma, C.; Sinquefield, S. A.; Shofner, M. L.; Nair, S. High-Performance Graphene Oxide Nanofiltration Membranes for Black Liquor Concentration. *ACS Sustain. Chem. Eng.* **2019**, *7* (17), 14915-14923. DOI: 10.1021/acssuschemeng.9b03113

(2) Shen, H. P.; Wang, N. X.; Ma, K.; Wang, L.; Chen, G.; Ji, S. L. Tuning Inter-Layer Spacing of Graphene Oxide Laminates with Solvent Green to Enhance Its Nanofiltration Performance. *J. Membr. Sci.* **2017**, *527*, 43-50, Article. DOI: 10.1016/j.memsci.2017.01.003.

(3) Flores-Chaparro, C. E.; Castilho, C. J.; Kulaots, I.; Hurt, R. H.; Rangel-Mendez, J. R. Pillared Graphene Oxide Composite as an Adsorbent of Soluble Hydrocarbons in Water: Ph and Organic Matter Effects. *J. Environ. Manage.* **2020**, *259*, 110044. DOI: 10.1016/j.jenvman.2019.110044
